# Esophageal Rhabdoid-Like Tumor: A Rare Disease With Aggressive Clinical Behavior

**DOI:** 10.3389/fsurg.2020.596010

**Published:** 2020-11-24

**Authors:** Andrea Lovece, Daniele Bernardi, Barbara Bruni, Emanuele Asti, Claudio Clemente, Luigi Bonavina

**Affiliations:** Division of General and Foregut Surgery, Department of Biomedical Sciences for Health, University of Milan, IRCCS Policlinico San Donato, San Donato Milanese, Italy

**Keywords:** esophagus, dysphagia, rhabdoid tumor, undifferentiated cancer, minimally invasive esophagectomy

## Abstract

**Background:** Malignant rhabdoid tumor is a kidney childhood tumor with aggressive clinical behavior and a wide spectrum of histologic, immunophenotypic, and cytogenetic findings. Extra-renal rhabdoid tumors have been reported in the brain, breast, liver, pancreas, bladder, vulva, prostate, and colon. To date, only nine cases of esophageal rhabdoid tumors have been described, all in patients over 50-year old. We add to the current literature the case of an esophageal, poorly differentiated rhabdoid tumor occurring in a young man.

**Case Report:** A 24-year-old man was referred for progressive dysphagia, retrosternal pain, nausea, and food regurgitation. Esophagogastroduodenoscopy showed an obstructing neoplastic lesion of the distal esophagus associated with Barrett's esophagus. Biopsies revealed undifferentiated esophageal cancer with epithelial morphology and immunohistochemistry positive for CK pan, CK 7 e CK 8-18. Minimally invasive esophagectomy and extended lymphadenectomy was performed. Histopathology showed a poorly differentiated tumor, with morphologic characteristics of rhabdoid tumor, central necrosis and transmural infiltration of the esophageal wall. Definitive immunohistochemistry was positive for vimentin, CD34, synaptophysin, and INI1.

**Conclusion:** Esophageal rhabdoid tumor is extremely rare and highly aggressive, with only few patients alive at 1 year follow-up, according to our review. Immunohistochemistry characterization is critical for diagnosis. Minimally invasive esophagectomy is an appealing and possibly less morbid option compared to open surgery. However, further research is needed to investigate the potential role of targeted immunotherapy.

## Background

Rhabdoid tumor, first described by Beckwith and Palmer in 1978 (1) as an uncommon variant of Wilms' tumor, is a rare clinical entity. Malignant rhabdoid tumor (MRT) of the kidney shows a wide spectrum of histologic, immunophenotypic, and cytogenetic features (2). Extra-renal MRT has been reported in the brain, breast, liver, pancreas, bladder, vulva, prostate, and colon (3). It occurs mostly in adults and has a highly malignant behavior and dismal prognosis (4). This malignancy has also been referred as carcinosarcoma, pseudosarcoma, or sarcomatoid carcinoma. In the early 2000, the undifferentiated esophageal carcinoma with prominent rhabdoid features was identified as a distinct pathological entity (5). The main histopathologic characteristic is the phenotypic heterogeneity, probably related to the origin from a primitive pluripotential cell (6). To date, only nine cases of esophageal MRT have been described in the literature, all in patients over 50-year old. We report a case of esophageal, poorly differentiated MRT in a young man.

## Case Presentation

**Figure 1:**
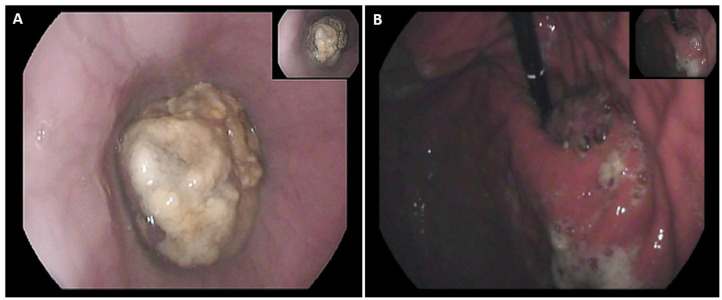
Endoscopic view: **(A)** proximal tumor margin; **(B)** retroflexed view of the cardia.

**Figure 2:**
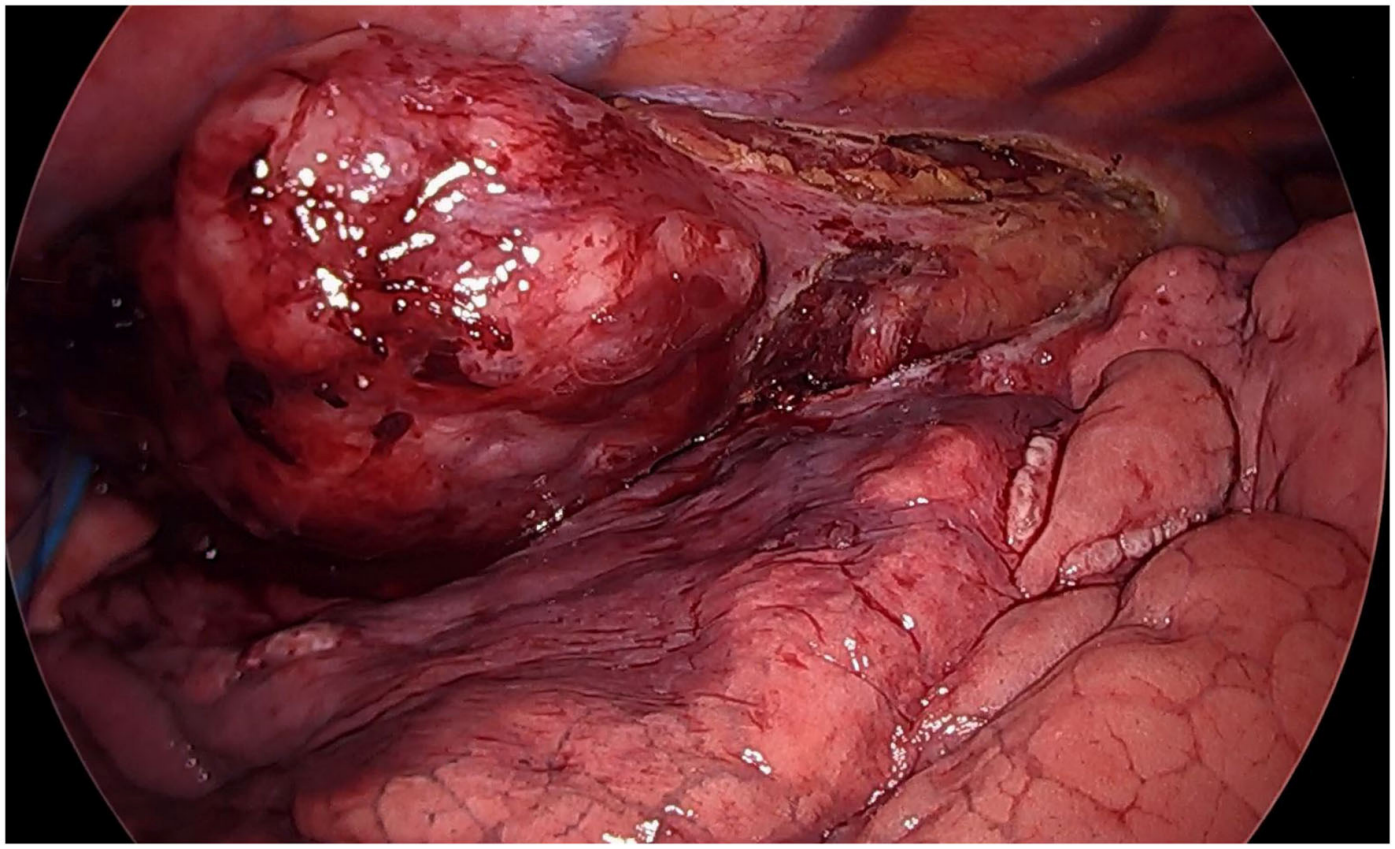
Thoracoscopic view (semi-prone position): huge neoplastic mass involving the infra-carenal esophagus.

A 24-year-old man, BMI 20, active smoker (1 pack/day), was referred to our outpatient clinic complaining of progressive dysphagia, retrosternal pain, nausea, and repeated episodes of melena food regurgitation. Past medical history was unremarkable, and there was no known familiarity for malignant tumors. Physical examination was normal, except for a marked weight loss (15 kg in 4 months), with no signs of anemia, jaundice, or palpable lymph nodes. An esophagogastroduodenoscopy showed a polypoid ulcerated lesion of the distal esophagus at 31–37 cm from the incisors, ulcerated and suspicion of Barrett's esophagus (Figure 1). Tracheobronchoscopy was negative. Biopsies revealed an undifferentiated esophageal cancer, with epithelial morphology and wide necrotic areas. Immunohistochemistry was positive for CK pan, CK 7 e CK 8-18, suggestive for carcinoma. Proliferation index (ki67) was classified as “high.” Computed-Tomography (CT) scan confirmed the presence of a 7 cm long and irregular stricture of the lower esophagus, with enlarged mediastinal nodes and no evidence of distant metastases. Positron-Emission Tomography (PET) scan showed focal radionuclide accumulation in the lower esophagus and mediastinal lymph nodes. The case was discussed at the local multidisciplinary tumor board, balancing potential risks and benefits of a neoadjuvant therapy on such an aggressive and advanced disease. A decision was taken to proceed with surgery, based on the patient's and family desire to avoid neoadjuvant therapy, considering also his absolute dysphagia and the high risk of tumor bleeding. Pre-operative work-up included ECG and respiratory function tests, which were normal. Blood test analysis showed hemoglobin 12.4 g/dL (n.v. 14–18 g/d/L), albumin 3.6 g/dL (n.v. 3.5–5.2 g/dL), Ca19.9 120 U/mL (n.v. <37 U/mL), CEA 12 ng/mL (n.v. <5 ng/mL). The patient was scheduled for a minimally invasive Ivor Lewis esophagectomy with celiac and mediastinal lymphadenectomy, and intrathoracic anastomosis (Figure 2). On post-operative day 4, the output of the thoracic drain became cloudy with abnormal amylase level. A CT scan revealed a small leakage from the esophago-gastric anastomosis, which was confirmed at endoscopy. The patient underwent right thoracotomy, washout of the pleural cavity, suture reinforcement of the anastomosis, and prophylactic endoscopic stenting. Subsequent postoperative course was uneventful, the stent was removed on postoperative day 21, and the patient was discharged home on day 29 eating a semisolid diet. Two months after surgery, the patient started to complain of low back pain; CT scan and magnetic resonance revealed multiple liver metastases (segments VI, VII, and IV), and bone metastases in the vertebral bodies and pelvis. Due to the compromised clinical conditions, no chemotherapy but only supportive treatment was proposed. The patient died of massive metastatic disease and pneumonia 3 months later.

### Histopathological Findings

**Figure 3:**
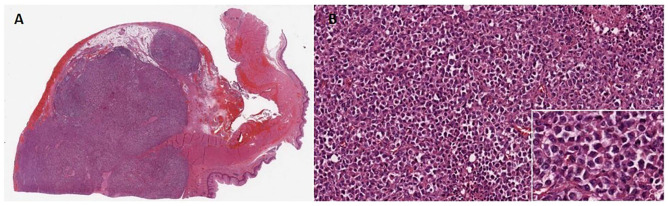
**(A)** Microscopic findings of the tumor at 5× magnification. Anaplastic large cells showing solid growth pattern and poorly cohesive growth. **(B)** Higher magnification (20×) view of the solid growth area. White box underlines at 100× magnification tumor cells exhibiting large nuclei with conspicuous nucleoli and eosinophilic “rhabdoid” cytoplasmic inclusions, while the nucleus is displaced eccentrically by the cytoplasmic inclusion body.

Macroscopically, a 6 cm long, whitish lesion was present in the distal esophagus with a 3 cm free proximal margin. The center of the tumor was located proximally to the esophagogastric junction. The lesion had a homogeneous surface and was infiltrating the mucosa. Thirty-four lymph nodes were also isolated from the specimen. Pathology revealed a poorly differentiated tumor, with morphologic characteristics of rhabdoid tumor, central necrosis and transmural infiltration of the esophageal wall (Figures 3A,B). Tumor metastases were found in 13 periesophageal and five perigastric lymph nodes.

Immunohistochemical staining of the specimen was positive for vimentin, CD34, synaptophysin, focal positivity for EMA and cytokeratin 8/18, whereas it was negative for cytokeratin PAN, cytokeratin 7, cytokeratin 19, S100, HMB45, tyrosinase, CD45, myeloperoxidase, CD138, myogenin, desmin, CD 3, CD 20, CD30, CD31, CD 43, CD 56, CD 68, CD 99, HHV8, BCL2, CD117, ACML, CM5.2, DOG1, p63, TTF1, chromogranin.

Proliferation index (Ki 67) was scored as “high.” The final diagnosis of neuroendocrine carcinoma with rhabdoid pattern was agreed with an expert consultant.

## Discussion

Extra-renal MRT are extremely rare tumors with a highly aggressive clinical behavior. They share the phenotype and immunohistochemistry features of renal MRT (7). It is still debated if they represent a specific histopathological entity or just a phenotype common to other poorly differentiated neoplasms. The ultrastructural features, first described by Haas et al. (8) and shared by the gastrointestinal MRT, consist of eccentrically located large nuclei with one or few prominent nucleoli and abundant cytoplasmatic PAS positive hyaline inclusions. Extra-renal MRT located in the gastrointestinal tract are extremely rare; Ueyama et al. (9), in 1993, reviewed 5,437 surgically resected gastric adenocarcinomas and found only four cases with rhabdoid features, representing <0.1% of all gastric carcinomas. Agaimy et al. (10), in 2014, reviewed 37 cases of gastrointestinal MRT reported in literature since 1989. Versteege et al. and Biegel et al. understood the common genetic signature of rhabdoid tumors: mutations involving the *SMARCB1 (INI1)* gene or, rarely, the *SMARCA4* gene, which encode proteins that are components of the chromatin remodeling complex SWI/SNF (11, 12). Since then, other cases of undifferentiated tumors with rhabdoid traits have been reported (13).

In Table 1, the main features of reported patients with esophageal MRT tumors, including our own case, are listed (6, 7, 13–17). In most (89%) patients, the tumor was located in the distal esophagus or gastroesophageal junction, and was associated with Barrett's esophagus in some cases. All patients have in common an immunohistochemistry positive for vimentin and different types of cytokeratin, as first described by Amrikachi et al. (14). Singhi et al. (13) reported that esophageal undifferentiated carcinoma and adenocarcinoma share a common origin from Barrett's esophagus, and may be considered two subtypes of the same lesion.

**Table 1:** List of all esophageal rhabdoid tumors reported in the literature.

**Author**	**Country**	**Year**	**No. pts**	**Age**	**Sex**	**Location**	**Barrett**	**Phenotype**	**POC**	**Transthoracic esophagectomy**	**DP (mos)**	**Primary metastatic location**	**1 year survival**
Amrikachi et al. (14)	USA	2002	2	61	M	Distal	NR	cytokeratin, vimentin	NR	No	<3 m	NR	no
				63	M	Distal	NR	vimentin, cytokeratin, synaptophysin, CD34	Uneventful	Yes	NR	NR	NR
Varghese et al. (7)	USA	2005	2	54	M	Distal	Yes	cytokeratin, vimentin	NR	NR	NR	Local lymphadenopathy	NR
				55	M	Distal	Yes	cytokeratin, vimentin	NR	NR	NR	Local lymphadenopathy	NR
Ng et al. (6)	China	2015	1	49	M	Middle third	No	vimentin, epithelial membrane antigen	Uneventful	Yes	3 m	Left cervical lymphadenopathy	No
Singhi et al. (13)	USA	2015	1	77	M	Distal	No	NR	NR	Yes	<3 m	Local lymphadenopathy	No
Kaechele et al. (15)	Germany	2015	1	57	M	NR	NR	NR	NR	No	<1 m	Liver	No
Ichimata et al. (16)	Japan	2019	1	81	M	GEJ	Yes	cytokeratin (CKAE1/AE3, CK8/18) vimentin, synaptophysin, neuron-specific enolase	Uneventful	Yes (MIE)	No	NR	Yes
Nagano et al. (17)	Japan	2019	1	67	M	GEJ	No	vimentin, CD34, cytokeratin (AE1/AE3)	Uneventful	Yes	No	Local lymphadenopathy	Yes
Present case	Italy	2020	1	24	M	Distal	Yes	vimentin, CD34, synaptophysin	Anastomotic leak	Yes (MIE)	<1 m	Local lymphadenopathy	No

*POC, post-operative course; DP, disease progression. GEJ, GastroEsophageal Junction; MIE, Minimally invasive esophagectomy*.

Despite the fact that most patients in this review had a locally advanced but non-metastatic tumor at diagnosis, all of them manifested disease progression within 3 months, and only two survived longer than 1 year. A SMARCB1 mutation was reported only in one patient (17). Preoperative imaging is not specific, and endoscopic biopsies rarely provide a complete immunohistochemical picture. This is confirmed in our case, as preoperative biopsies revealed an undifferentiated tumor but not its rhabdoid features. We underline the importance of extensive tissue sampling to allow a wider immunophenotypic study in case of a clinically aggressive esophageal tumor presentation in a young patient. Varghese et al. (7), indeed, underlined the importance of EUS-guided FNA (Fine-Needle Aspiration) to confirm the tumor phenotype. So far, surgical resection is the only available therapeutic option when the tumor appears non-metastatic and resectable. The role of neoadjuvant and adjuvant therapies for MRT of the gastrointestinal tract is unknown. Horazdovsky et al. (18) analyzed a total of 167 cases of extra-renal, extra-central nervous system MRT and found that surgery and chemotherapy with actinomycin were the only factors associated with a significant reduction of the risk of death. Amrikachi et al. (14), in their review of GI rhabdoid tumors, reported that 75% of patients died within 10 months from presentation due to disease progression. Only recently, targeted immunotherapy has become a new possible therapeutic option in MRT. Abro et al. (19) reported that blockade of the programmed cell death 1/programmed cell death ligand 1 (PD-1/PD-L1) immune checkpoint pathway could be one of the most promising approaches for these patients.

## Conclusions

We reported an extremely rare case of esophageal MRT occurring in a 24-year old patient. To date, no effective neoadjuvant or adjuvant treatment is available, leaving surgery as the only palliative option for non-metastatic and resectable tumors. Minimally invasive esophagectomy, as reported in this study, is a more attractive and possibly less morbid option compared to the open approach. The role of targeted immunotherapy is promising, but further studies are needed.

## Patient Perspective

The patient was well aware of the rarity of his tumor and of the limited chances of cure. He took active part to each decision regarding his therapeutic process. He provided a written informed consent for the publication of his case in order to help a better understanding of the disease and to contribute to a more effective treatment in the future.

## Data Availability Statement

The original contributions presented in the study are included in the article/supplementary materials, further inquiries can be directed to the corresponding author/s.

## Ethics Statement

Written informed consent was obtained from the individual(s) for the publication of any potentially identifiable images or data included in this article.

## Author Contributions

AL searched the literature and was a major contributor in writing the manuscript. DB participated at the operation and was a contributor in writing and reviewing the manuscript. BB performed the histological examination of the esophagus. LB operated the patient, reviewed the manuscript and was a contributor in writing it. All authors contributed to the article and approved the submitted version.

## Conflict of Interest

The authors declare that the research was conducted in the absence of any commercial or financial relationships that could be construed as a potential conflict of interest.

## Abbreviations

MRT: Malignant Rhabdoid Tumor
CT: Computed Tomography
PET: Positron-Emission Tomography
FNA: Fine-Needle Aspiration
PD-1/PD-L1: programmed cell death 1/programmed cell death ligand 1.

## References

[1] Beckwith JB Palmer NF. Histopathology and prognosis of Wilms' tumor: results from the first national Wilms' tumor study. Cancer. (1978) 41:1937–48. 10.1002/1097-0142(197805)41:5<1937::AID-CNCR2820410538>3.0.CO;2-U 206343

[2] Palmer NF Sutow W. Clinical aspects of the rhabdoid tumor of the kidney: a report of the National Wilms' Tumor Study Group. Med Pediatr Oncol. (1983) 11:242–5. 10.1002/mpo.2950110407 6310357

[3] Lupi G Jin R Clemente C. Malignant rhabdoid tumor of the vulva: a case report and review of the literature. Tumori. (1996) 82:93–5. 10.1177/030089169608200120 8623515

[4] Uwineza A Gill H Buckley P. Rhabdoid tumor: the Irish experience 1986-2013. Cancer Genet. (2014) 207:398e402. 10.1016/j.cancergen.2014.05.015 25085603

[5] Ota S Crabbe DCG Tuan TN Triche TJ Shimada H. Malignant rhabdoid tumor. A study with two established cell lines. Cancer. (1993) 71:2862–72. 10.1002/1097-0142(19930501)71:9<2862::AID-CNCR2820710930>3.0.CO;2-D 8385567

[6] Ng W Leong H Ma K Yip W Suen W. Malignant rhabdoid tumour of the oesophagus: a case report. J Clin Pathol. (2003) 56:713–4. 10.1136/jcp.56.9.713 12944560 PMC1770065

[7] Varghese L Stanley MW Lucido M. Esophageal carcinoma with a rhabdoid phenotype: a case report of diagnosis by endoscopic ultrasound-guided fine-needle aspiration. Diagn Cytopathol. (2005) 33:407–11. 10.1002/dc.20362 16299741

[8] Haas JE Palmer NF Weinburg AG Beckwith JB. Ultra-structure of malignant rhabdoid tumor of kidney: a distinct renal tumor of the children. Hum Pathol. (1981) 12:646–57. 10.1016/S0046-8177(81)80050-0 7275104

[9] Ueyama T Nagai E Yao T. Vimentin-positive gastric carcinomas with rhabdoid features. A clinicopathologic and immunohistochemical study. Am J Surg Pathol. (1993) 17:813–9. 10.1097/00000478-199308000-00006 7687828

[10] Agaimy A Rau T Hartmann A. SMARCB1 (INI1)-negative rhabdoid carcinomas of the gastrointestinal tract: clinicopathologic and molecular study of a highly aggressive variant with literature review. Am J Surg Pathol. (2014) 38:910–20. 10.1097/PAS.0000000000000173 24503755

[11] Versteege I Sevenet N Lange J Rousseau-Merck MF Ambros P Handgretinger R. Truncating mutations of hSNF5/INI1 in aggressive pediatric cancer. Nature. (1998) 394:203–6. 10.1038/28212 9671307

[12] Biegel JA Zhou JY Rorke LB. Germ-line and acquired mutations of INI1 in atypical teratoid and rhabdoid tumors. Cancer Res. (1999) 59:74–9. 9892189

[13] Singhi A Seethala R Nason K. Undifferentiated carcinoma of the esophagus: a clinicopathological study of 16 cases. Hum Pathol. (2015) 46:366–75. 10.1016/j.humpath.2014.11.021 25582499 PMC4384179

[14] Amrikachi M Ro J Ordonez N Ayala A. Adenocarcinomas of the gastrointestinal tract with prominent rhabdoid features. Ann Diagn Pathol. (2002) 6:357–63. 10.1053/adpa.2002.36657 12478486

[15] Kaechele V Vogelpohl J Boeck W. Malignant extrarenal rhabdoid tumour (MERT) with liver metastases as a rare cause of an esophageal tumor in a 57 years old patient. Z Gastroenterol. (2015) 53:660–3. 10.1055/s-0034-1399694 26167696

[16] Ichimata S Aoyagi D Takehana T. A case of large cell neuroendocrine carcinoma exhibiting rhabdoid features in the esophagogastric junction. Pathol Intern. (2019) 69:481–7. 10.1111/pin.12807 31237049

[17] Nagano H Izumi T Kawahara E. SMARCB1- and vimentin-positive esophageal carcinoma with undifferentiated components, rhabdoid features, and a good prognosis: a case report. Surg Case Rep. (2019) 5:8. 10.1186/s40792-019-0562-4 30649642 PMC6335233

[18] Horazdovsky R Manivel JC Cheng EY. Surgery and actinomycin improve survival in malignant rhabdoid tumor. Sarcoma. (2013) 2013:315170. 10.1155/2013/315170 23431248 PMC3574752

[19] Abro B Kaushal M Chen L. Tumor mutation burden, DNA mismatch repair status and checkpoint immunotherapy markers in primary and relapsed malignant rhabdoid tumors. Pathol Res Pract. (2019) 215:15239. 10.1016/j.prp.2019.03.023 31047727

